# Intravenous Thrombolysis in Acute Ischemic Stroke with Active Cancer

**DOI:** 10.1155/2017/4635829

**Published:** 2017-06-04

**Authors:** Ki-Woong Nam, Chi Kyung Kim, Tae Jung Kim, Sang Joon An, Kyungmi Oh, Sang-Bae Ko, Byung-Woo Yoon

**Affiliations:** ^1^Department of Neurology, Seoul National University Hospital, Seoul, Republic of Korea; ^2^Department of Neurology, Korea University Guro Hospital and Korea University College of Medicine, Seoul, Republic of Korea

## Abstract

Ischemic stroke patients with active cancer are known to have poor clinical outcomes. However, the efficacy and safety of intravenous alteplase (IV t-PA) in this group are still unclear. In this study, we aimed to evaluate whether stroke patients with cancer had poor clinical outcomes after use of IV t-PA. We reviewed ischemic stroke patients with active cancer treated with isolated IV t-PA between April 2010 and March 2015 at three national university hospitals from the registry for ischemic stroke in Korea. The clinical outcomes of early neurological deterioration (END), hemorrhagic transformation, in-hospital mortality, 3-month modified Rankin scale (mRS), the National Institutes of Health Stroke Scale (NIHSS) discharge score, and duration of hospitalization were compared. We enrolled a total of 12 patients, and the cohort showed poor outcomes including 4 (33%) END events, 7 (58%) hemorrhagic transformations, 3 (25%) in-hospital mortality cases, and 7 (58%) poor mRS (3–6) scores. Additionally, the cryptogenic stroke group (*n* = 6) more frequently had high mRS scores (*P* = 0.043) as well as tendencies for frequent END events, hemorrhagic transformations, in-hospital mortality cases, and higher discharge NIHSS scores without statistical significance. In conclusion, ischemic stroke patients with active cancer, especially those with a cryptogenic mechanism, showed poor clinical outcomes after use of IV t-PA.

## 1. Introduction

Ischemic stroke in cancer patients, with a frequent occurrence of up to 15%, has been recently studied [1]. These patients have complicated stroke mechanism by conventional stroke mechanisms (e.g., large-artery atherosclerosis, small-vessel disease, and cardioembolism) and cancer-specific mechanisms (e.g., hypercoagulability, tumor embolism, and nonbacterial thrombotic endocarditis) [2]. Previously, patients who experienced stroke with cryptogenic mechanism, who had no evidence of conventional mechanisms, were proven to be more closely related to those with stroke with cancer-specific mechanisms (especially hypercoagulability) and had poorer clinical outcomes [1, 3–9].

Intravenous alteplase (IV t-PA) is a well-known treatment option to recover from poststroke disability during the acute period. However, its efficacy and safety in patients with active cancer have not been well addressed due to its complex stroke mechanisms [6]. With longer life-expectancy due to improved cancer treatments, we needed to assess the exact prognosis and identify the high-risk subset after use of IV t-PA in ischemic stroke patients with active cancer.

In this study, we evaluated whether ischemic stroke patients with active cancer had poor clinical outcomes after use of IV t-PA. In addition, we also aimed to evaluate the impact of stroke mechanisms (e.g., conventional versus cryptogenic mechanisms) on the clinical outcomes after use of IV t-PA.

## 2. Methods

### 2.1. Study Population

**Figure 1:**
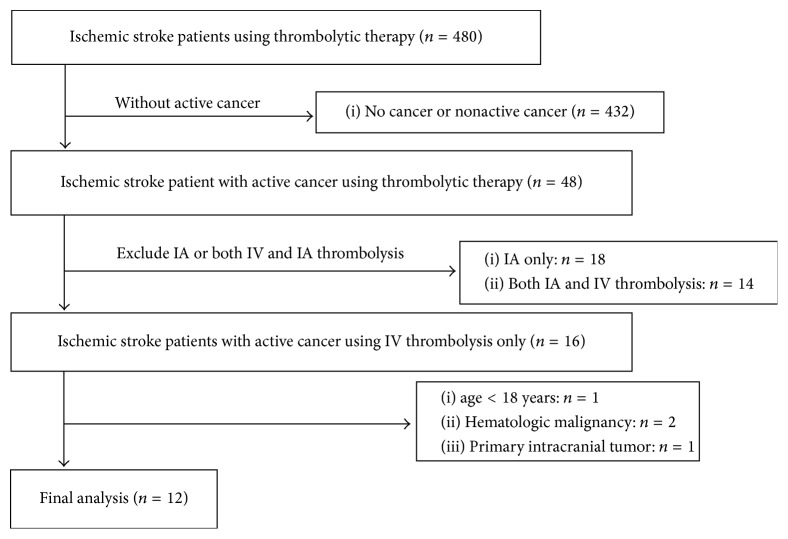
Patient selection for the study.

We retrospectively reviewed medical records from the consecutively enrolled stroke registry for ischemic stroke patients with active cancer who visited three national university hospitals (Seoul National University Hospital, Seoul National University Bundang Hospital, and Seoul Metropolitan Government-Seoul National University Boramae Medical Center) in Korea between April 2010 and March 2015. Active cancer was defined as any diagnosis, recurrence, metastasis, and progression of cancer within 6 months of enrollment [6]. Among the cases, we extracted the subpopulation that was treated with IV t-PA. Patients using both IV t-PA and intra-arterial thrombectomy were excluded as to evaluate the sole effects of IV t-PA for the study. In addition, we also excluded patients under the age of 18 years and those who had a hematologic malignancy or a primary intracranial tumor which are known to have different stroke mechanisms [6]. Finally, a total of 12 patients remained for the analysis (Figure 1). This study was approved by the institutional review board (IRB) of Seoul National University Hospital (H-1610-036-797) and designed according to the STrengthening the Reporting of OBservational studies in Epidemiology (STROBE) guidelines.

### 2.2. Clinical Assessment

We collected demographic, clinical, and cardiovascular risk factors, including the presence of hypertension, diabetes, hyperlipidemia, current smoking, use of alcohol, history of stroke, initial stroke severity, mechanisms of stroke, blood pressure (BP), initial antithrombotics taken, and dose of t-PA [3]. The initial stroke severity was assessed using the National Institutes of Health Stroke Scale (NIHSS) by well-trained neurologists. The mechanisms of stroke were classified using the Trial of Org 10172 in Acute Stroke Treatment (TOAST) classification. We, then, dichotomized the stroke mechanisms into conventional (large-artery atherosclerosis, small-vessel occlusion, and cardioembolism) and cryptogenic mechanisms [6]. BP was assessed for initial systolic BP and diastolic BP. Initial antithrombotics were divided into low-molecular weight heparin, warfarin, antiplatelet agent, and no treatment. Data regarding the patient's cancer were also assessed including cancer type, systemic or brain metastasis, and venous thromboembolism (e.g., deep-vein thrombosis, pulmonary embolism).

We assessed the clinical outcomes in variable aspects. We evaluated early neurological deterioration (END), hemorrhagic transformation, hospitalization duration, discharge NIHSS scores, in-hospital mortality events, and the 3-month modified Rankin scale (mRS) scores. END was defined as an increase of ≥1 in the motor NIHSS score or ≥2 in the total NIHSS score [3].

### 2.3. Radiological Assessment

All patients underwent brain magnetic resonance imaging (MRI) within 4 hours of admission using a 3.0-Tesla MR scanner (Achieva 3.0 T; Philips, Eindhoven, Netherlands). We dichotomized the initial diffusion-weighted image lesion into single territory lesions and multiple territory lesions [7]. Laboratory results, including C-reactive protein, D-dimer, prothrombin time, and activated partial thromboplastin time were assessed within 24 hours of admission.

### 2.4. Statistical Analysis

All statistical analyses were conducted using SPSS version 22 (IBM SPSS, Chicago, IL, USA). We presented continuous variables as the mean ± SD when data were normally distributed, while the others were presented as the median + interquartile range. Student's* t*-test or Mann–Whitney* U* test were used for continuous variables, and the chi-squared test or Fisher's exact test were used for categorical variables. All variables with* P* < 0.05 were considered significant in the statistical analyses.

## 3. Results

We enrolled a total of 12 ischemic stroke patients with active cancer who were treated with IV t-PA (mean age of 69 years, visit time 1 [0.5–1.75] hours, median NIHSS scores 10 [7–19], Table 1). Among them, 6 patients were classified as having a conventional stroke mechanism, and the remaining with a cryptogenic mechanism. In clinical outcomes, the cohort had 4 (33%) END events, 7 (58%) hemorrhagic transformations, 3 (25%) in-hospital mortalities, and 7 (58%) poor mRS (3–6) scores. The duration of hospitalization was 12 [9–25] days.

**Figure 2:**
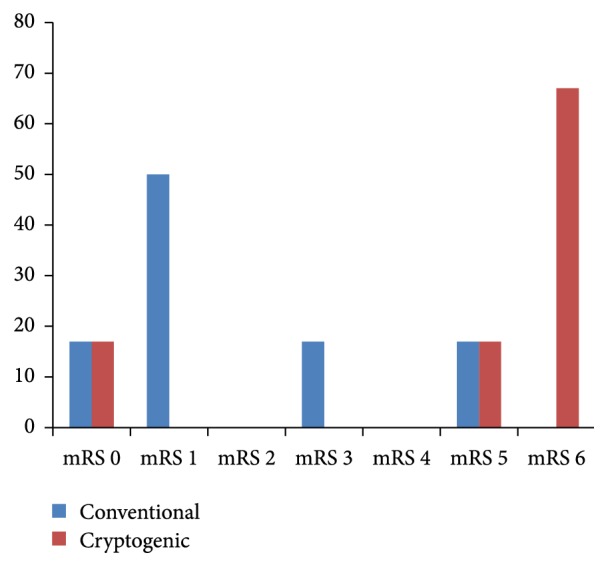
Distributions of 3-month mRS scores between patients that experienced stroke with conventional and cryptogenic mechanisms.* P* for trend < 0.043 in linear-by-linear association.

None of the demographic, clinical, cardiovascular, laboratory, or radiological variables were significantly different between the conventional and the cryptogenic groups (Table 2). However, in clinical outcomes, the cryptogenic group had higher 3-month mRS scores (*P* = 0.043, Figure 2). The cryptogenic group also had a tendency of more frequent END events, hemorrhagic transformations, in-hospital mortality cases, and higher discharge NIHSS scores without statistical significance. Three in-hospital mortality cases occurred only in the cryptogenic group, and the causes of death were pneumonia aggravation, myocardial infarction, and stroke recurrence.

## 4. Discussion

In this study, ischemic stroke patients with active cancer showed poor outcomes after use of IV-tPA. Furthermore, cryptogenic stroke mechanisms seemed to be related to poor outcomes.

According to a previous study, ischemic stroke patients with cancer were proven to be as safe as noncancer patients in a large population study, showing 12% in-hospital mortality and 6% intracerebral hemorrhage cases [10]. However, this study included cancer-associated stroke with relatively heterogeneous traits (e.g., including hematologic malignancy, nonactive cancer with stably controlled states, treated by both IV-tPA and intra-arterial thrombectomy). Thus, the pure outcomes of cancer-associated stroke may be hard to interpret. Additionally, there have also been two case-series studies that reported on safety and hazardous events after use of t-PA in ischemic stroke with cancer [11, 12]. The cases that resulted in hazardous events occurred in patients with newly diagnosed cancer and high elevated D-dimer levels with nonbacterial thrombotic endocarditis, similar to our participants [11, 12]. In contrast, patients with fair outcomes were those with nonactive cancer status, defined as being stable after operation or in complete remission with regular treatment [12]. In this study, we attempted to collect data from patients with relatively homogenous cancer-related stroke, and these patients showed poorer outcomes than previous studies in variable clinical aspects (e.g., in-hospital mortality, hemorrhagic transformation, and END), although the 3-month mRS scores were not significantly different [13].

Additionally, the mechanisms of stroke in this group seemed to play a role in the clinical outcomes. Those with cryptogenic stroke showed more frequent END events, hemorrhagic transformation, in-hospital mortality, and a higher number of discharge NIHSS scores and 3-month mRS scores despite these patients having the same initial NIHSS scores as the other group. We already knew that cryptogenic stroke in cancer patients had a worse outcome than conventional stroke in the clinical outcomes of stroke [14]. However, the result of having worse clinical outcomes with cryptogenic stroke after use of t-PA is interesting. We hypothesize that having a more advanced cancer and an impaired coagulation cascade might play a role.

This study is the first report on clinical outcomes after using t-PA in homogeneous cancer-related stroke and the impact of the stroke mechanisms on clinical outcomes. However, there are some caveats to this study. This study was designed as a three-center, uncontrolled, retrospective study. A small sample size limited the statistical power, despite considering the difficulty of collecting homogenous traits of this group. There was also a possibility of selection bias as a result of the retrospective.

In conclusion, ischemic stroke patients with active cancer showed poor clinical outcomes after use of IV t-PA. As the cryptogenic group showed worse outcomes, a more careful observation of this group should be recommended.

## Figures and Tables

**Table 1:** Brief profile of participants in the study.

Number/sex/age, y	Cancer type	t-PA dose	Initial D-dimer	Mechanism	Initial NIHSS	END	3m mRS	Hemorrhagic transformation	Initial treatment
1/F/64	Lung	0.9	26.8	Cryptogenic	20	N	5	Y	Enoxaparin
2/M/61	Pancreas	0.9	5.11	Cryptogenic	7	Y	6	Y	Enoxaparin
3/F/74	Pancreas	0.9	3.7	Cryptogenic	13	Y	6	Y	No treatment
4/M/61	Lung	0.6	2.01	Cryptogenic	7	N	0	N	Enoxaparin
5/M/76	Lung	0.6	3.91	Cryptogenic	15	Y	6	Y	No treatment
6/M/82	Gastric	0.6	20	Cryptogenic	7	N	6	Y	Enoxaparin
7/M/70	Gastric	0.9	11.1	CE	7	N	1	Y	Warfarin
8/M/81	Colon	0.6	0.82	LAD	19	Y	3	Y	Antiplatelet
9/M/64	Colon	0.6	0.73	LAD	3	N	0	N	Antiplatelet
10/M/67	Lung	0.6	1.77	LAD	19	N	1	N	Antiplatelet
11/F/56	Cervical	0.6	0.16	LAD	5	N	1	N	Antiplatelet
12/F/76	Lung	0.9	8.28	CE	24	N	5	Y	Enoxaparin

**Table 2:** Baseline characteristics and clinical outcomes between conventional and cryptogenic stroke mechanisms.

	Conventional (*n* = 6)	Cryptogenic (*n* = 6)	*P* value
Time delay to visit, h [IQR]	1 [1-2]	1 [0-1]	0.624
Age, y [SD]	69 ± 9	70 ± 9	0.899
Sex, male %	4 (67)	4 (67)	1.000
Hypertension, %	3 (50)	1 (17)	0.545
Diabetes, %	2 (33)	0 (0)	0.455
Hyperlipidemia, %	1 (17)	2 (33)	1.000
Current smoking, %	2 (33)	1 (17)	1.000
Alcohol, %	4 (67)	1 (17)	0.242
History of stroke, %	1 (17)	0 (0)	1.000
Venous thrombosis, %	0 (0)	2 (33)	0.455
Cancer type, %			0.688
Lung	2 (33)	3 (50)	
Gastric	1 (17)	1 (17)	
Colorectal	2 (33)	0 (0)	
Hepatobiliary	0 (0)	2 (33)	
Genitourinary	1 (17)	0 (0)	
Systemic metastasis, %	1 (17)	5 (83)	0.080
Brain metastasis, %	0 (0)	3 (50)	0.182
Initial NIHSS [IQR]	13 [5–19]	10 [7–15]	0.935
SBP, mmHg [SD]	130 ± 27	151 ± 28	0.213
DBP, mmHg [SD]	77 ± 23	84 ± 12	0.479
Initial antithrombotics, %			0.476
Low-molecular weight heparin	1 (17)	4 (67)	
Warfarin	1 (17)	0 (0)	
Antiplatelet agent	4 (67)	0 (0)	
No treatment	0 (0)	2 (33)	
Intravenous alteplase dose, %			1.000
0.6 mg/kg	4 (67)	3 (50)	
0.9 mg/kg	2 (33)	3 (50)	
Initial DWI lesion			0.061
Single territory	6 (100)	2 (33)	
Multiple territory	0 (0)	4 (67)	
D-dimer, *µ*g/mL [IQR]	1.30 [0.73–8.28]	4.51 [3.70–20.0]	0.109
CRP, mg/dL [IQR]	0.03 [0.03–0.11]	2.79 [0.38–12.20]	0.085
PT, INR [SD]	1.09 ± 0.05	1.15 ± 0.20	0.497
aPTT, sec [SD]	34.1 ± 4.6	31.5 ± 9.8	0.578
Hospital stay, day [IQR]	16 [9–28]	12 [6–17]	0.420
Discharge NIHSS [IQR]	4 [2–7]	28 [8–42]	0.063
Early neurological deterioration, %	1 (17)	3 (50)	0.545
Hemorrhagic transformation, %	2 (33)	5 (83)	0.242
In-hospital mortality, %	0 (0)	3 (50)	0.182
3m mRS, %			0.043
0	1 (17)	1 (17)	
1	3 (50)	0 (0)	
2	0 (0)	0 (0)	
3	1 (17)	0 (0)	
4	0 (0)	0 (0)	
5	1 (17)	1 (17)	
6	0 (0)	4 (67)	

We used Student's *t*-test or Mann–Whitney *U* test for continuous variables, while chi-square test or Fisher's exact test was used for categorical variables.
